# Success of a urinary catheter insertion team in reducing urinary infections in the ICU

**DOI:** 10.1186/cc14690

**Published:** 2015-09-28

**Authors:** Deianira Regagnin, Debora Schettini da Silva Alves, Luciana Reis Guastelli

**Affiliations:** 1 Department of Critically Ill Patients, Hospital Israelita Albert Einstein, São Paulo, SP, Brazil

## Introduction

About 8-21 % of hospital infections in ICUs are urinary [1,2], 80 % of them being associated with the use of urinary catheters [3]. Several studies show that the early removal of urinary catheters reduces the rate of urinary tract infection. However, critically ill patients who require this device do not have the option to remove. For this group, the best preventive measure seems to be educative activity for the nursing staff responsible for the insertion and manipulation of this device.

## Objective

To create a team of professionals trained in the insertion of urinary catheters and to organize actions aimed at reducing the rate of urinary tract infection associated with urinary catheters in the ICU.

## Methods

Prospective study conducted for 12 months in the ICU. Started in July 2013, the intervention program involved the creation of a qualified team for the insertion of urinary catheter and the creation of audits to stimulate the removal of inappropriate urinary catheters and assess the process of inserting these devices. The obtained results were compared with the 12 months preceding the beginning of the interventions.

## Results

**Figure 1:**
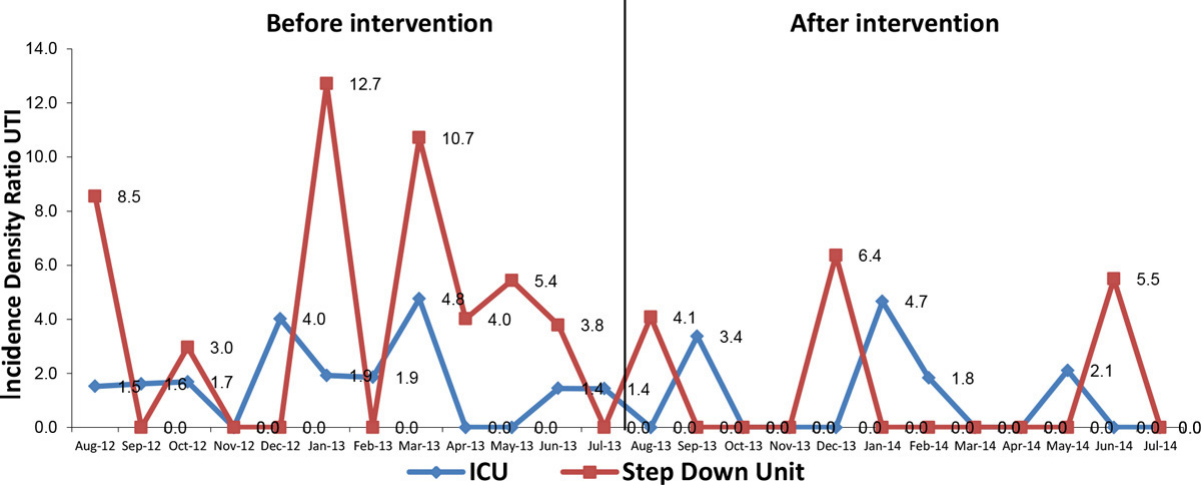
**Incidence density ratio of urinary tract infections before and after interventions**.

**Figure 2:**
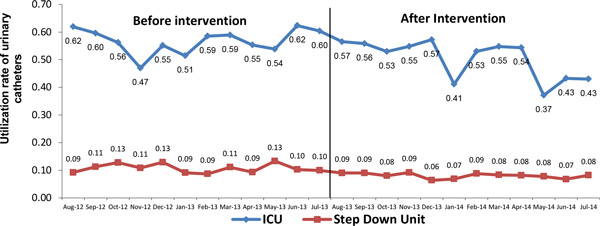
**Utilization rate of urinary catheters before and after interventions**.

Comparison between August 2012-July 2013 and August 2013-July 2014 (Table 1, Figures 1 and 2) shows that there was a fall of 57.2 % (2.4-1.0, *p *= 0.040) in the rate of urinary tract infection associated with a urinary catheter and a reduction of 13.4 % (from 0.24 to 0.21, *p *= 0.001) in the utilization rate of urinary catheters. In the 12 months after intervention (August 2013-July 2014) the percentage of compliance of technical insertion of urinary catheter was 97 % and the inappropriate removal rate of urinary catheters was 85 % (Table 2).

**Table 1:** Incidence density ratio urinary tract infection and utilization ratio urinary catheter before and after interventions according to the location.

Place	Ratio	Time	Mean	DP	Median	Min	Max	Mean reduction (%)	*p *value
									
ICU	IDR UTI	Before	1.69	1.47	1.57	0.00	4.76	40.9	0.286
		After	1.00	1.62	0.00	0.00	4.66		
	UR	Before	0.57	0.05	0.57	0.47	0.62	11.3	**0.016**
		After	0.50	0.07	0.54	0.37	0.57		
SDU	IDR UTI	Before	4.01	4.52	3.38	0.00	12.71	66.9	0.084
		After	1.33	2.45	0.00	0.00	6.37		
	UR	Before	0.11	0.02	0.11	0.09	0.13	25.0	**<0.001**
		After	0.08	0.01	0.08	0.06	0.09		
ICU + SDU	IDR UTI	Before	2.39	1.89	2.08	0.00	6.59	57.2	**0.040**
		After	1.02	1.07	1.20	0.00	3.23		
	UR	Before	0.24	0.02	0.25	0.21	0.26	13.4	**0.001**
		After	0.21	0.02	0.22	0.16	0.23		

Student *t *test

*SDU *step down unit, *IDR UTI *incidence density ratio of urinary tract infection,

*UR *utilization ratio of urinary catheter

**Table 2:** Results of audited items between August 2013 and July 2014.

	ICU	SDU	ICU + SDU
Number of patients observed	9571	25,716	35,287
Number of patients with IDC	4752	2370	7122
% Urinary catheter appropriate	92	86	90
% Urinary catheter inappropriate	10	16	12
% Discontinued inappropriate IDC	97	70	85
Number of insertions of IDC audited	803	280	1083
% Proper techniques for insertion of IDCs	99	95	97

*IDC *indwelling urinary catheters; *SDU *step down unit

## Conclusion

The results show that low-cost educational interventions can reduce urinary infections and provide more security for patients in ICUs.
